# Value-based medicine: concepts and application

**DOI:** 10.4178/epih/e2015014

**Published:** 2015-03-04

**Authors:** Jong-Myon Bae

**Affiliations:** Department of Preventive Medicine, Jeju National University School of Medicine, Jeju, Korea

**Keywords:** Evidence-based medicine, Evidence-based practice, Quality of life, Value of life, Decision support techniques

## Abstract

Global healthcare in the 21st century is characterized by evidence-based medicine (EBM), patient-centered care, and cost effectiveness. EBM involves clinical decisions being made by integrating patient preference with medical treatment evidence and physician experiences. The Center for Value-Based Medicine suggested value-based medicine (VBM) as the practice of medicine based upon the patient-perceived value conferred by an intervention. VBM starts with the best evidence-based data and converts it to patient value-based data, so that it allows clinicians to deliver higher quality patient care than EBM alone. The final goals of VBM are improving quality of healthcare and using healthcare resources efficiently. This paper introduces the concepts and application of VBM and suggests some strategies for promoting related research.

## INTRODUCTION

Evidence-based medicine (EBM) became the prevailing medical paradigm in the post-1990s era. At the core of EBM is clinical decision-making based on the best available research evidence rather than a clinician’s expertise to minimize the uncertainties of clinical examinations [1,2]. In an effort to help clinicians utilize the newest and highest quality research evidence, The Evidence-Based Medicine Working Group [3] suggested a five-level grading system for assessing the quality of evidence [1].

However, a different viewpoint soon followed that the best evidence alone is not always sufficient for making the most rational clinical judgments and decisions. Accordingly, clinicians’ expertise pertaining to the particulars of the circumstances at hand and patients’ values should also be considered in the decision-making process [4]. Incorporating this point of view, the definition of EBM was modified to “the integration of best research evidence with clinical expertise and patient values” [5]. Subsequently, the likelihood of being helped or harmed index was developed, which reflects patients’ expectations regarding the benefits and risks of treatment options. Despite such efforts, the EBM paradigm still poses limitations in actively incorporating health-related quality of life (HRQoL) [6] and patients’ preferences into the realm of medicine and public health [7].

Despite the advantage of producing the highest quality evidence, the efficacy of randomized clinical trials fails to provide a true indication of clinical effectiveness. In this light, patient-centered care (PCC) started to gain momentum, where patients as medical consumers participate in the decision-making process (shared decision-making) and evaluation of treatment outcomes (patient-reported outcomes) [8]. Furthermore, PCC corresponds to comparative effectiveness research (CER) and its goals of achieving maximum benefit per cost [9]. PCC is also in accordance with the principles of medical ethics [10]. Finally, PCC strives to improve healthcare quality through integration of patient values in medical care [11].

According to this backdrop, Brown et al. [12] elaborated on the three major trends of EBM, PCC, and CER in 21st century healthcare. As the movement to actively integrating patient preferences (values) into clinical practice takes hold, value-based medicine (VBM) has emerged in order to stress a medicine based on values as well as research evidences. In early years, it was also referred to as “humanized medicine” because its core concept of integrating scientific research and patient values is congruent to the principles of medical ethics [13]. The present paper aims to introduce the concept of VBM to the Korean healthcare system and make recommendations about its potential applications.

## BODY

### Value-based medicine: definition and objectives

The term “value-based” was first introduced by a team of researcher led by Dr. Brown at the Center for Value-Based Medicine at Pennsylvania State University [14]. The team defined VBM as “the practice of medicine incorporating the highest level of evidence-based data with the patient-perceived value conferred by healthcare interventions for the resources expended” [15,16]. In the above sentence, three major components of VBM are highlighted [17]. First, EBM principles are thoroughly selected based on the best research evidence available and applied as treatment options. Second, patients’ values are converted into measurable utility values to facilitate the integration. Third, the cost-utility level expected from selecting a particular treatment option is the basis for decision-making. In other words, the ultimate goal pursued by VBM is to provide cost effective, science-based healthcare that incorporates patient values [7,14]. Petrova et al. [13] suggested 10 principles that can be applied in clinical settings to achieve these goals.

### Values and utility in healthcare

The dictionary definition of “value” is “relative worth, utility, or importance” [9]. Its operational definition varies depending on the agents who assign values such as in patient and social values [13,14,18]. Considering that VBM focuses on patient values, the definition of values here concerns (1) increased life expectancy or (2) increased HRQoL [7]. Increased life expectancy can easily be measured by concrete indexes such as survival rate. However, measuring HRQoL improvements poses yet another challenge [19].

Numerous tools have been developed to measure HRQoL that have been applied in various fields [20]. These tools can be classified into function-based and preference-based tools [14], and the measurement based on preference is referred to as “utility” [15]. In other words, values that judiciously reflect patient preferences are connected to utility by HRQoL [9,16]. On a side note, “utility” in the context of healthcare is based on von Neumann-Morgenstern’s utility theorem, which concerns individuals’ decision-making processes amid uncertainties [21]. In a similar vein, utility in our context can be interpreted as the level to which an individual’s choice reflected upon trade-off between the potential benefits and risks under circumstances in which uncertainties abound [16,22].

### Utility value and utility score

Utility value is obtained by measuring patient preference levels, with “0” indicating death, by far the worst case scenario in healthcare, and “1” indicating perfect health [17,23,24]. To measure the utility values that correspond to various disease conditions, the standard gamble, time trade-off, and rating scale methods were developed [22,25]. Among them, the time trade-off method is the most commonly used in VBM research for its easy comprehensibility, validity, and repeatability [6,9,14,15,23]. To compare some outcomes, the measuring method should be verified [16] because these methods have been developed out of different theoretical backgrounds [26].

The utility value is the benefit level at a specific point in time (age), which means it can vary over time [27]. That is, it is necessary to weight time points in different health states, which vary with age [25]. The utility value does not consider life expectancy; therefore, incorporating the additional element of life expectancy would render a more faithful healthcare value [16]. Reflecting this viewpoint, quality-adjusted life years (QALY), disability-adjusted life years, healthy-year equivalents, and health-adjusted life expectancy have been developed [15] whose calculated indexes are called utility scores [22]. QALY, which is calculated by multiplying year(s) of life by utility value(s), is the most widely used value index in healthcare and related research efforts [22]. In VBM, the total value of medical intervention is calculated using a decision tree model and QALY [2,15]. Links to more information on the principles behind the development of QALY, calculation method, advantages, and disadvantages can be found in the references section of this paper [17,24].

### Value-based medicine application steps

Brown et al. [15] suggested seven steps for the research process. However, I suggest restructuring the process into four steps, which are shown in Table 1 along with the research methodologies and concepts applied to each step.

Steps 1 and 2, in which evidence is searched and evaluated upon the construction of a clinical problem, are identical to the flow of Ask, Acquire, and Appraisal on EBM 5A steps [1,28]. When implementing VBM for the purpose of selecting a medical intervention, it is important to clarify the items pertaining to comparison (comparators) among the population, intervention, comparators and outcome parameters (PICO) applied in Step 1. Once evidences about economic feasibility were acquired in Step 2, its validity, impact, and applicability are evaluated for utilization [29,30]. In this step, it must be verified whether the information required for the cost analysis and decision tree in Step 3 – cost, utility value, and utility level – can be acquired, and the result should be reflected in the next step.

Step 3 concerns the cost-utility analysis (CUA), which is the core of VBM research [31,32]. CUA, a method of economic evaluation, used to concern a concept that clearly differed from what was handled by cost-effective analysis (CEA) [24]. Recently, however, it tends to be viewed as a method incorporated into CEA [33]. Nevertheless, QALY is an index that was developed to reflect utility in the first place. Therefore, it makes sense to classify the research concerning money per QALY as CUA for data comparisons and valid interpretations [31,34]. CUA involves separate calculation processes of cost and utility. In Steps 3a and 3b, a decision tree is used to calculate the total utility value and QALY as in the provided example [7,32,35]. For information about the types of costs and discount rates needed in Step 3c, please refer to the references pertaining to economic evaluation [14,16,24,36].

Regarding the discount rate, it should be noted that an identical discount rate is used for both the cost and utility value gained and that the most recent year’s data are not applicable [15,37]. In recent years, a new index called ‘return on investment’ has been suggested for the purpose of financial assessment [38]. In Step 3d, the cost-utility ratio, as the ultimate product of CUA, is calculated and expressed as the money per QALY unit [16, 23,39,40]. Until the mid-2000s, decisions were made based on a fixed money amount per QALY unit [41,42]. However, this has recently been criticized for being irrational [43,44]. The use of incremental CUR, which compares the disparity between treatment alternatives, appears to be more suitable [15].

Finally, in Step 4, a sensitivity test is performed to eliminate uncertainties and enable reasonable data interpretation [2]. Through this process, outcome stability is verified even if the assumptions or applied utility levels change [24]. If reporting the above results in a paper, please refer to the suggestions by Siegel et al. [45] and the Task Force on Principles for Economic Analysis of Health Care Technology [46].

### Usefulness and limitations of value-based medicine

VBM actively embraces patient values and quality of life, which were overlooked by EBM [13,17]. VBM has great potential to improve the quality of healthcare [47] since it is both congruent with the principles of medical ethics [13] and capable of reducing the uncertainties of clinical decisions [22]. Additionally, VBM is in accordance with the goals of health economics [14,24] since it facilitates an efficient allocation of resources by prioritizing the options by maximum utility per cost [14,15,23,32].

Nevertheless, VBM has its limitations since it concerns values [15,16,22,23]. First, the various values pursued by various involved agents, such as medical service providers, insurance providers, and policy makers, are likely to conflict with each other. Second, although patient values are prioritized, patients may exhibit a number of different values, which may change over time. Third, the availability of a variety of treatment options for accompanying diseases may not be guaranteed. Fourth, there is no standardized database available regarding utility values, which can provide an indication about quality of life. Fifth, the threshold set for the cost-utility ratio is unclear. Sixth, comparisons are impossible between countries with different economic structures and healthcare delivery systems.

## CONCLUSION AND SUGGESTIONS

As seen to date, VBM expands on EBM by incorporating CUA to provide healthcare that reflects patient values (preferences) [9,14,22,23]. The ultimate goals of VBM are improved healthcare quality and effective and efficient utilization of healthcare resources [15].

To help the Korean healthcare system successfully actualize the goals of VBM, I offer a few strategic recommendations. First, collaborative clinical epidemiologic research is needed to establish a utility value database centered on major diseases that incorporates the time trade-off methods [1,14]. The patient QALY by disease and QALY league tables can be calculated and established only upon acquiring such a database [48]. In the process, participants who represent patient groups will have to be selected and reference case values will have to be collected [15,35]. Second, a healthcare system should be embraced that actively adopts clinical decision-making processes based on patient preferences. This means that the current common practice of offering a hustled explanation about treatment options and then obtaining written patient consent must be re-examined to offer healthcare that can actively incorporate patient values under uncertain circumstances [2]. To achieve this goal, decision aids that improve patients’ understanding their diseases personally will require development [49,50]. Last, to the greatest extent possible, a consistent application of standardized VBM research is recommended [14,17,32].

## Figures and Tables

**Table 1.** Four steps of applying value-based medicine

Step	Action	Related methods and concepts
1	Asking an answerable question	P opulation, intervention, comparators and outcome parameters
2	Confirming the best evidence	comparators and outcome parameters
3	Gathering the numerical values	Cost-utility analysis
3a	Measuring the utility value	Time trade-off
3b	Calculating the total value	Decision tree and QALY
3c	Estimating costs	Cost analysis with discounting
3d	Calculating cost-utility ratios	Cost per QALY
4	Handling uncertainty	Sensitivity analysis

Modified from Brown et al. Evidence-based to value-based medicine. Chicago: AMA Press; 2005 [15].

QALY, quality-adjusted life years.
