# Supplementary material for: Value-based medicine: concepts and application
Source: Epidemiol Health. 2015 Mar 4;37:e2015014. doi: 10.4178/epih/e2015014 (PMC4398974; doi:10.4178/epih/e2015014)
Supplement: Supplementary file 1 [file epih-37-e2015014-supplementary.pdf]

<연구방법론>

가치기반의학: 개념 및 적용

Value-Based Medicine: Concepts and Application

Abstract

The global health care in the 21st century is characterized as evidence-based medicine (EBM), patient-centered care, and cost effectiveness. The EBM recommends that the clinical decision should be made with integrating patient's preference with the best evidences and physician's experiences. The Center for Value-Based Medicine suggested the Value-Based Medicine (VBM) as the practice of medicine based upon the patient-perceived value conferred by an intervention. In other words, VBM starts with the best evidence-based data and converts the data to patient value-based form. VBM allows clinicians to deliver higher quality patient care than EBM alone. The final goals of VBM are to improve quality of healthcare and to use healthcare resources efficiently. This paper introduces the concepts and application of VBM and suggests some strategies for activating the VBM research.

Key words: Evidence-based Medicine, Evidence-based practice, Quality of life, Value of life, Decision support techniques

1. 서론 : 가치기반의학의 제기 배경

임상진료의 불확실성을 최소화하기 위하여 임상의사의 경험에 따른 직관보다는 '최선 (the best)'의 근거에 기반하여 임상결정을 하자는 근거중심의학 (Evidence-based Medicine, 이하 EBM)은 1990년대 이후 현대 의학의 핵심 패러다임으로 자리잡게 되었다[1-2]. 그리고 최선의 근거를 적용하기 위하여, The Evidence-based Medicine Working Group[3]은 기존의 연구결과들을 5가지 등급으로 분류하는 것을 제안하였고, 최신의 정보를 포함하여 가장 높은 수준의 근거를 활용하는 것이 바로 EBM의 지향점이다[1].

그러나 임상 판단 및 결정을 위해서는 최선의 근거를 적용하는 것도 중요하지만, 개별적인 임상상황에 대한 의사의 임상경험과, 환자의 가치 (value)도 같이 반영해야 한다는 주장이 곧 이어 제기되었다[4]. 이에 따라 EBM 정의가 최선의 의학적 근거를 임상가의 전문성과 환자의 가치를 통합하는 것 (The integration of best research evidence with clinical expertise and patient values)이라고 수정되었다[5]. 그리고 선택할 의료기술의 득실에 대한 환자의 생각을 반영하여 득실지표 (the Likelihood of being helped or harmed index)를 개발하였다[2]. 이런 노력에도 불구하고 환자의 선호도를 포함하여 보건관련 삶의 질 (Health-related Quality of Life, 이하 HRQoL) [6]까지 보건의료영역에 적극적으로 반영하기 위해서는 EBM 패러다임만으로는 한계가 있다[7].

한편, 가장 높은 수준의 근거를 만들어 내는 무작위배정 임상시험 (randomized clinical trial)으로 얻어내는 효능 (efficacy)은 실제적인 임상 효과 (effectiveness)를 제대로 반영하지 못하기 때문에, 의료소비자인 환자가 치료 선택에 직접 참여하고 (shared decision making), 치료결과를 직접 평가하는 (patient-reported outcome) 환자중심의 진료 (patient-

centered care, 이하 PCC)가 강조되고 있다[8]. 더군다나 PCC는 비용대비 최대효과를 얻으려는 비교효과연구 (Comparative Effectiveness Research, 이하 CER)의 지향점과 상응하고[9] 의료윤리 원칙들과 부합하면서[10], 환자가치의 반영은 곧 보건의료의 질 향상을 달성하는 전략의 하나로 간주되고 있다[11].

위와 같은 추세 속에 Brown et al. [12]이 언급한 21세기 보건의료의 3가지 흐름 – EBM, PCC, CER –에 부응하기 위해서, 환자의 선호도로 표현되는 가치를 임상진료에 적극 개입하자는 움직임이 본격화되는 과정에서 근거뿐만 아니라 가치에 기반한 의학을 강조하기 위하여 가치기반의학 (Value-based Medicine, 이하 VBM; 국내에서는 Evidence-based를 '근거중심'으로 번역하여 널리 사용하고 있는 가운데, 최근 중요성이 부각된 Patient-centered를 '환자중심'이라고 부르고 있다. 다른 단어를 같은 용어로 중첩하여 사용하는 것은 개념상 혼선을 불러온다는 점에서 'based'란 단어에 충실하고자 '기반'이란 용어를 사용한다. 이를 계기로 '근거중심'도 '근거기반'이란 용어로 전환하여 사용하기를 제안한다.)이 제기된 것이다. 초기에 과학적 근거에 바탕하면서, 환자의 가치를 반영하려는 개념이 의료윤리와 부합되면서 한때 'humanized medicine'이란 용어를 사용한 적도 있다[13]. 본 원고는 이런 배경을 가진 VBM를 국내 보건의료계에 소개하고, 적용에 대한 제언들을 하고자 한다.

2. 본론 : 가치기반의학 관련 개념 정의와 적용 방법론

가. 가치기반의학 정의와 목표

'Value-based'란 용어는 미국 Pennsylvania 대학에 소재한 the Center for Value-Based Medicine의 Brown 박사 연구팀이 처음 제안하였다[14]. 해당 연구팀이 제시한 VBM 정의는 보건의료 서비스 제공을 받기 위해 지불을 결정하는 환자의 가치와 최선의 근거기반 자료를 통합하려는 진료의학 (Value-based medicine is the practice of medicine incorporating the highest level of evidence-based data with the patient-perceived value conferred by healthcare interventions for the resources expended.이다[15,16].

이상의 문장에서 VBM은 3가지 주요 요소들을 강조하고 있다[17]. 첫째, 최상의 근거를 활용하여 특정 시술을 선택한다는 점에서 EBM의 방법론을 온전히 적용한다. 둘째, 환자가 추구하는 가치를 반영하기 위하여 보건의료에 맞는 효용 가치 (utility value)로 변환하고 계량화 한다. 셋째, 특정 시술의 시행에 따라 지불할 비용 대비 얻게 될 효용 수준을 선택의 기준으로 삼겠다는 것이다. 따라서 과학적 근거를 기반으로 하고, 환자의 가치를 추구하여 비용대비 최대 효용이 보장된 진료를 하겠다는 것이 VBM의 지향 목표이다[7,14]. 이 목표를 달성하기 위하여 Petrova et al. [13]은 진료현장에 적용할 10가지 원칙 – 근거기반의 진료, 진료상담 기술 활용, 의사결정 공동 참여, 의료전달체계 고려 등-을 제시하였다.

나. 보건의료에서의 가치와 효용 개념

'가치'란 용어에 대하여 사전적 정의는 '상대적 값어치, 효용, 혹은 중요성 (relative

worth, utility, or importance)'이며[9], 가치부여의 주체에 따라 환자의 가치[14], 사회적 가치 [18] 등으로 분류하여 조작적 정의를 하고 있다[13]. 그런데 VBM에서는 환자의 가치에 집중하고 이를 진료결정에 반영한다는 점을 염두에 두고 보건의료 차원에서 가치를 정의한다면 (1) 생존기간을 연장시키거나 (2) 보건관련 삶의 질 (HRQoL)을 높이는 것이다[7]. 이 중 생존기간 연장에 대하여는 생존율 등의 지표로 쉽게 계량화할 수 있지만, HRQoL 향상을 측정하는 것은 또 다른 도전이 된다[19]

지금껏 HRQoL을 측정하기 위한 여러 도구들이 개발되어 다양한 영역에 적용되었다[20]. 이들 도구들을 기능 (function)에 바탕 한 것과 선호도 (preference)에 바탕 한 측정법으로 양분하는데[14], 이중 선호도에 바탕 하여 측정한 결과물을 특히 효용 (utility)이라 한다[15]. 즉, 환자의 선호도를 충실히 반영한 가치 개념은 HRQoL이란 가교를 통해 효용 개념과 연결된다[9,16]. 한편, 보건의료에 있어 효용 개념은 불확실한 상황에서 개인의 의사결정에 관한 von Neumann-Morgenstern 효용경제이론을 배경으로 하고 있다[21]. 이에 따라 효용은 불확실성 속에서 예상되는 득과 실에 관한 저울질 (trade-off)을 통해 개인의 의사결정이 반영된 수준으로 해석한다[16,22].

다. 효용가치 (utility value)와 효용점수 (utility score)

보건의료 영역에 있어서 최악의 결과인 사망을 0점으로, 완전한 건강을 1점으로 간주한 다음, 특정 질병을 가진 환자의 선호도 수준을 측정하여 얻어낸 결과수치를 효용가치 (utility value)라 한다[17,23,24]. 그리고 각종 질병상태에 따른 효용가치를 측정하기 위하여 Standard Gamble, Time Trade-off, Rating Scaling의 3가지 방법이 개발되어 있다[22,25]. 이 중 시간교환법 (Time trade-off)이 응답자가 이해하기 쉽고, 타당성과 재현성이 높다는 장점 때문에 VBM 연구에서 가장 많이 활용하고 있다[6,9,14,15,23]. 그렇지만 각 방법들은 서로

다른 이론적 배경에서 개발되었기 때문에[26], 사용한 측정법을 먼저 확인한 후 동일한 측정법 결과들에서만 상호 비교가 가능하다[16].

한편 효용가치는 특정 시점 (연령)에서의 효용수준을 의미하며, 시간이 흐른다면 그 효용가치는 변화하게 된다[27]. 즉 연령변화에 따른 건강수준변화를 반영한 가중치를 반영할 필요가 있다[25]. 더군다나 효용가치가 다루지 않은 생존기간 연장도 추가로 반영한다면 보다 충실하게 보건의료 가치를 갖게 될 것이다[16]. 이런 점을 감안하여 Quality-adjusted life years (QALY), Disability-adjusted life years (DALY), Healthy-years equivalents (HYE), Health-adjusted life expectancy (HALE) 등이 개발되었으며[15], 그 산출지표 값을 효용점수 (utility score)라 한다[22]. 이중 시간구간별 효용가치의 곱으로 산출되는 QALY가 보건의료 영역의 가치를 대변하는 대표적인 지표로, 관련 연구에서 가장 잘 활용되고 있다[22]. VBM은 결정나무 (Decision tree)와 QALY 산출을 통해 의료기술의 총 가치 (the total value)을 산출한다[2,15]. 본 원고에서는 QALY 개발 원리, 산출법, 장단점 등은 참고문헌을 소개하는 것으로 대신 한다[17,24]

라. 가치기반의학 수행단계

Brown et al. [15] 은 연구수행을 7 단계로 제시하고 있지만, 저자는 4 단계로 재분류하고 각 단계마다 적용하는 연구 방법론과 개념을 Table 1로 제시한다.

문제 설정 후 관련 근거들의 검색 및 평가를 하는 1, 2 단계는 EBM의 근거생성 5A 중 Ask, Acquire, Appraisal 흐름과 동일하다[1,28]. 특히 의료기술의 선택을 목적으로 VBM이 수행되는 경우에는 1단계에서 적용하는 PICO 중 비교 (Comparator)에 대한 항목을 보다

분명히 하는 것이 중요하다. 그리고 2단계에서 경제성 평가와 관련한 논문들이 확보되었다면 타당성 (validity), 영향 (impact), 적용가능성 (applicability)의 차원에서 이를 평가하고 활용할 근거를 확보한다[29-30]. 이 과정에서 3단계에서 이루어질 비용분석과 결정나무분석에 사용할 정보들 – 비용, 효용가치, 효용수준 등-에 대한 수집 가능성을 확인하고 다음 단계에 이를 반영토록 한다.

3 단계는 비용-효용분석 (cost-utility analysis, 이후 CUA)을 하는 것으로, VBM 연구의 핵심 활동이다[31,32]. 경제성 평가 (Economic Evaluation) 방법의 하나인 CUA는 비교-효과분석 (cost-effective analysis, CEA)과 분명히 다른 개념을 다루는 독립된 방법론이었으나 [24], 최근 들어 CEA에 속한 것으로 보는 경향이 있다[33]. 그렇지만 QALY는 본래 효용을 담기 위해 개발한 지표인 만큼, 결과의 상호비교와 타당한 해석을 위해 money per QALY로 발표되는 연구를 CUA라 정의하자는 주장이 합당하다[31,34]. CUA 수행에는 비용과 효용을 각각 산출하는 작업이 필요한데, 효용가치를 알아내는 3a와 3b 단계에서는 결정나무를 이용하여 총효용가치와 QALY를 산출하는데, 실제 수행 예를 참고토록 한다[7,32,35]. 3c 단계인 비용 산출에 있어 비용의 종류와 할인율 (discounting)에 대하여는 경제성 평가 관련 문헌을 참조하기 바란다[14,16,24,36]. 할인율에 있어 짚고 갈 점은 비용뿐만 아니라 얻게 될 효용 가치 (utility value gained)에 대하여도 동일한 할인율을 적용한다는 점과, 최근 1년은 적용하지 않는다는 것이다[15,37]. 최근에는 보건정책학적인 재정 평가를 위하여 투자 회수률 (return-on-investment, ROI)이란 지표도 제시되었다[38]. 3d 단계로 CUA의 최종 산출물인 비용-효용비 (cost-utility ratio)를 산출하며 money per QALY 단위로 제시한다 [16,23,39,40]. 그런데 2000년 중반까지 단위 QALY 당 일정 금액을 기준으로 의사결정을 해왔으나[41,42], 최근에는 비합리적이란 지적을 받고 있다[43,44]. 대신 비교대상이 되는 의료기술에 비하여 얼마나 차이가 있는가를 보는 Incremental CUR을 활용하는 것이 합당하겠다

[15].

마지막 4 단계는 타당한 결과 해석을 위하여 불확실성을 배제할 목적으로 민감도 분석을 시행하는 것이다[2]. 이 과정을 통해 연구에 적용된 가정이나 대입한 효용 수준이 변화하더라고 연구 결과가 안정성을 갖는가를 확인한다[24]. 그리고 이상의 결과들을 논문으로 보고할 때는 Siegel et al. [45]과 Task Force on Principles for Economic Analysis of Health Care Technology [46]의 제안들을 참조한다.

마. 가치기반의학의 유용성과 한계점

VBM은 EBM으로 담을 수 없는 환자의 가치를 적극 수용한다는 점에서 환자의 삶의 질에 대한 적극적인 고려를 하므로[13,17], 의료윤리 원칙에도 부합하고[13] 임상결정의 불확실성을 줄일 수 있어[22] 진료의 질적 수준을 향상시킬 수 있다[47]. 또한 비용대비 효용을 극대화하는 선택을 우선하기에 제한된 의료자원의 효율적인 할당을 이루게 해준다는 점에서[14,15,23,32] 보건경제학의 지향점과 일치한다[14,24].

그러나 VBM은 가치를 다루는 만큼 여러 한계점을 가진다[15,16,22,23]. 첫째, 환자 이외 다른 주체들 – 의료서비스 제공자, 의료보험 공급자, 의료정책 결정자 등 –의 다양한 가치와 상충할 가능성이 높다. 둘째, 환자의 가치를 우선한다고 해도, 환자들 간에도 다양한 가치가 표출할 수 있고 시간에 따라 변화한다. 셋째, 동반질환에 대한 진료 선택의 다양성을 보정할 수 없다. 넷째, 삶의 질을 알아낼 수 있는 효용가치에 대한 표준화 데이터베이스가 없다. 다섯째, 비용 대비 효용의 판단기준점 (threshold)이 불분명하다. 여섯째, 경제구조와 의료전달체계가 다른 나라간 상호비교가 불가능하다.

3. 결어 및 제언

앞서 살펴본 바에 따르면, VBM은 EBM을 기반한 가운데 환자의 가치 (선호도)를 반영한 진료를 행하기 위하여 CUA분석을 적용하는 것이다[9,14,22,23]. 이를 통해 의료의 질을 높이고 보건의료의 효율적 사용을 도모하는 것이 VBM의 궁극적 목표이다[15].

그러나 국내 진료환경에서 VBM의 취지를 달성하기 위해서 몇 가지 전략적 제언을 하고자 한다. 첫째, 질병부담이 높은 주요 질병을 중심으로 시간교환법 등을 적용하여 효용가치 (utility value)의 데이터베이스를 만드는 임상역학적 공동연구가 필요하다[1,14]. 이 값들이 확보되어야만 해당 질병별 환자들의 QALY를 산출하고 나아가 QALY league tables 도 작성할 수 있기 때문이다[48]. 이때 환자군을 대표하는 대상자를 선정하고 이들로부터 reference case value를 얻어내는 작업이 이루어져야 할 것이다[15,35]. 둘째, 개별 환자의 선호도에 맞춘 임상결정이 이루어지는 진료환경 시스템을 적극 도입해야 한다. 시술에 대한 간단한 설명으로 서면 동의서를 받는 현재의 사무행정적 수준에서 벗어나, 불확실한 상황 발생에 대한 환자의 가치를 능동적으로 반영할 수 있도록 해야 할 것이다[2]. 이를 위해서는 환자 스스로 질병에 대한 이해를 높이게 해주는 의사결정조력도구 (decision aids) 등의 개발이 동반되어야 할 것이다[49,50]. 셋째, 가급적이면 표준화된 VBM 연구방법에 따라서 일관성 있는 적용을 할 필요가 있다[14,17,32].

감사의 글

이 논문은 2014학년도 제주대학교 학술진흥연구비 지원사업에 의하여 연구되었음

References

1. Bae JM, Park BJ, Ahn YO. Perspectives of clinical epidemiology in Korea. J Korea Med Assoc 2013;56:718-723. (korean)

2. Bae JM. The clinical decision analysis using decision tree. Epidemiol Health 2014;36:e2014025.

3. Evidence-Based Medicine Working Group. Evidence-based medicine. A new approach to teaching the practice of medicine. JAMA 1992;268:2420-2425.

4. Haynes RB, Devereaux PJ, Guyatt GH. Clinical expertise in the era of evidence-based medicine and patient choice. ACP J Club 2002;136:A11-A14.

5. Straus SE, Glasziou P, Richardson WS, Haynes RB. Evidence-based medicine. How to practice and teach it. 4th ed. Edinburgh; Churchill Livingstone; 2011. p 1-12.

6. Stein JD, Brown MM, Brown GC, Hollands H, Sharma S. Quality of life with macular degeneration: perceptions of patients, clinicians, and community members. Br J Ophthalmol 2003;87:8-12.

7. Brown MM, Brown GC, Brown HC, Irwin B, Brown KS. The comparative effectiveness and cost-effectiveness of vitreoretinal interventions. Curr Opin Ophthalmol 2008;19:202-207.

8. Bae JM. Global trends in the use of nationwide big data for solving healthcare problems. J Korean Med Assoc 2014;57:386-390.

9. Brown MM, Luo B, Brown HC, Brown GC. Comparative effectiveness: its role in the healthcare system. Curr Opin Ophthalmol 2009;20:188-194.

10. Yoo SH, Lee JY, Lee KH, Lee IH, Bae JM. Ethical principles and practice guidelines concerning the usage of public database for medical researches. J Korean Med Assoc 2013;56:1031-1038.

11. Bae JM, Jung SE, Park BJ. A case study of Institute of Medicine for developing strategies of National Academy of Medicine of Korea. Health Policy Forum. 2014;12:104-110. (korean)

12. Brown GC, Brown MM, Sharma S. Health care in the 21st century: evidence-based medicine, patient preference-based quality, and cost effectiveness. Qual Manag Health Care 2000;9:23-31.

13. Petrova M, Dale J, Fulford BK. Values-based practice in primary care: easing the tensions between

individual values, ethical principles and best evidence. Br J Gen Pract 2006;56:703-709.

14. Brown MM, Brown GC. Update on value-based medicine. Curr Opin Ophthalmol 2013;24:183-189.

15. Brown MM, Brown GC, Sharma S. Evidence-based to value-based medicine. Chicago: AMA Press; 2005. p.5-7, 125-149, 151-181, 193-206, 207-217, 267-279, 319-324.

16. Brown MM, Brown GC, Sharma S, Landy J. Health care economic analyses and value-based medicine. Surv Ophthalmol 2003;48:204-223.

17. Brown GC, Brown MM, Sharma S, Brown H, Smithen L, Leeser DB, et al. Value-based medicine and ophthalmology: an appraisal of cost-utility analyses. Trans Am Ophthalmol Soc 2004;102:177-185.

18. Barthel HJ. Evidence-based medicine: the way to cost-effective, quality medical care. US Army Med Dep J. 2007:63-68.

19. Unruh ML, Weisbord SD, Kimmel PL. Health-related quality of life in nephrology research and clinical practice. Semin Dial 2005;18:82-90.

20. Vetter TR. A primer on health-related quality of life in chronic pain medicine. Anesth Analg 2007;104:703-718.

21. Torrance GW. Preferences for health outcomes and cost-utility analysis. Am J Manag Care 1997;3 Suppl:S8-S20.

22. Birch S, Ismail AI. Patient preferences and the measurement of utilities in the evaluation of dental technologies. J Dent Res 2002;81:446-450.

23. Brown MM, Brown GC, Sharma S. Value-based medicine and vitreoretinal diseases. Curr Opin Ophthalmol 2004;15:167-172.

24. Kim CM. Health economics and outcomes research. Korean J Fam Med 2009;30:577-587. (korean)

25. Burrows C, Brown K. QALYs for resource allocation: probably not and certainly not now. Aust J Public Health 1993;17:278-286.

26. Drummond MF, Sculpher MJ, Torrance GW, O'Brien BJ, Stoddart GL. Methods for the economic evaluation of health care programmes. 3rd ed. New York: Oxford University Press; 2005. p.140-147.

27. Bravo Vergel Y, Sculpher M. Quality-adjusted life years. Pract Neurol 2008;8:175-182.

28. Sackett DL. Clinical epidemiology. what, who, and whither. J Clin Epidemiol 2002;55:1161-1166.

29. O'Brien BJ, Heyland D, Richardson WS, Levine M, Drummond MF. Users' guides to the medical literature. XIII. How to use an article on economic analysis of clinical practice. B. What are the results and will they help me in caring for my patients? Evidence-Based Medicine Working Group. JAMA 1997;277:1802-1806.

30. Vogel B, Gilbert SM, Boylston Herndon J, Dahm P. Advanced topics in evidence-based urologic oncology: economic analysis. Urol Oncol 2011;29:454-461.

31. Brown GC, Brown MM, Sharma S. Health care economic analyses. Retina 2004;24:139-146.

32. Brown MM, Brown GC, Lieske HB, Lieske PA. Preference-based comparative effectiveness and cost–effectiveness: a review and relevance of value-based medicine for vitreoretinal interventions. Curr Opin Ophthalmol 2012;23:163-174.

33. Griebsch I, Coast J, Brown J. Quality-adjusted life-years lack quality in pediatric care: a critical review of published cost-utility studies in child health. Pediatrics 2005;115:e600-e614.

34. Birch S, Gafni A. Cost effectiveness/utility analyses. Do current decision rules lead us to where we want to be? J Health Econ 1992;11:279-296.

35. Gupta OP, Brown GC, Brown MM. A value-based medicine cost-utility analysis of idiopathic epiretinal membrane surgery. Am J Ophthalmol 2008;145:923-928.

36. Brown GC. Vision and quality-of-life. Trans Am Ophthalmol Soc 1999;97:473-511.

37. Weinstein MC, Siegel JE, Gold MR, Kamlet MS, Russell LB. Recommendations of the Panel on Cost-effectiveness in Health and Medicine. JAMA 1996;276:1253-1258.

38. Brown MM, Brown GC, Lieske HB, Lieske PA. Financial return-on-investment of ophthalmic interventions: a new paradigm. Curr Opin Ophthalmol 2014;25:171-176.

39. Brown MM. Health care economic analyses. Curr Opin Ophthalmol 2003;14:117-121.

40. Chapman RH, Berger M, Weinstein MC, Weeks JC, Goldie S, Neumann PJ. When does quality-adjusting life-years matter in cost-effectiveness analysis? Health Econ 2004;13:429-436.

41. Laupacis A, Feeny D, Detsky AS, Tugwell PX. How attractive does a new technology have to be to warrant adoption and utilization? Tentative guidelines for using clinical and economic evaluations. CMAJ 1992;146:473-481.

42. Schackman BR, Gold HT, Stone PW, Neumann PJ. How often do sensitivity analyses for economic parameters change cost-utility analysis conclusions? Pharmacoeconomics 2004;22:293-300.

43. Brown MM, Brown GC, Sharma S, Garrett S. Evidence-based medicine, utilities, and quality of life. Curr Opin Ophthalmol 1999;10:221-226.

44. Braithwaite RS, Meltzer DO, King JT Jr, Leslie D, Roberts MS. What does the value of modern medicine say about the $50,000 per quality-adjusted life-year decision rule? Med Care 2008;46:349-356.

45. Siegel JE, Weinstein MC, Russell LB, Gold MR. Recommendations for reporting cost-effectiveness analyses. Panel on Cost-Effectiveness in Health and Medicine. JAMA 1996;276:1339-1341.

46. Task Force on Principles for Economic Analysis of Health Care Technology. Economic analysis of health care technology. A report on principles. Ann Intern Med 1995;123:61-70.

47. Brown GC. Value-based medicine: the new paradigm. Curr Opin Ophthalmol 2005;16:139-140.

48. Robinson R. Cost-utility analysis. BMJ 1993;307:859-862.

49. O'Connor A. Using patient decision aids to promote evidence-based decision making. ACP J Club 2001;135:A11-A12.

50. Bae JM. Academic strategies based on evidence-practice gap. Hanyang Med Rev 2015;35:3-8 (korean)

Table 1. Four steps of applying the value-based medicine*

Step	Actions	Related methods & concepts
1	Making an Answerable Question	PICO
2	Confirming the best evidences	Searching & Appraisal
3	Gathering the numerical values	Cost-Utility analysis
3a	Measuring the utility value	Time Trade-off
3b	Calculating the total value	Decision Tree & QALY
3c	Estimating Costs	Cost analysis with discounting
3d	Calculating cost-utility ratios	Money per QALY
4	Handling the Uncertainty	Sensitivity analysis

* Modified from Reference [15]
